# Biofilm-associated proteins: from the gut biofilms to neurodegeneration

**DOI:** 10.1080/19490976.2025.2461721

**Published:** 2025-02-03

**Authors:** Jaione Valle

**Affiliations:** Microbial Biotechnology Department, Instituto de Agrobiotecnología, CSIC-Gobierno de Navarra, Mutilva, Navarra, Spain

**Keywords:** α-synuclein, amyloid, biofilm, biofilm-associated protein, gut microbiota

## Abstract

Human microbiota form a biofilm with substantial consequences for health and disease. Numerous studies have indicated that microbial communities produce functional amyloids as part of their biofilm extracellular scaffolds. The overlooked interplay between bacterial amyloids and the host may have detrimental consequences for the host, including neurodegeneration. This work gives an overview of the biofilm-associated amyloids expressed by the gut microbiota and their potential role in neurodegeneration. It discusses the biofilm-associated proteins (BAPs) of the gut microbiota, maps the amyloidogenic domains of these proteins, and analyzes the presence of *bap* genes within accessory genomes linked with transposable elements. Furthermore, the evidence supporting the existence of amyloids in the gut are presented. Finally, it explores the potential interactions between BAPs and α-synuclein, extending the literature on amyloid cross-kingdom interactions. Based on these findings, this study propose that BAP amyloids act as transmissible catalysts, facilitating the misfolding, accumulation, and spread of α-synuclein aggregates. This review contributes to the understanding of complex interactions among the microbiota, transmissible elements, and host, which is crucial for developing novel therapeutic approaches to combat microbiota-related diseases and improve overall health outcomes.

## Introduction

**Figure 1.**
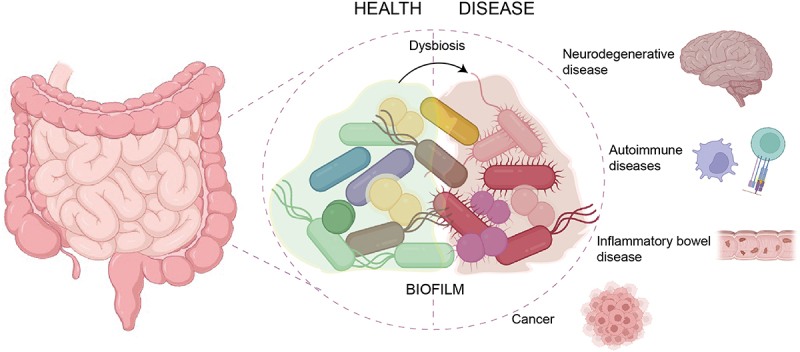
Role of intestinal biofilm in health and disease. Gut microbiota live as biofilms and provide many vital functions for the host. Alterations of the microbiota promote the growth of pathobionts. Biofilms formed by pathobionts may cause and progress various diseases, including: neurological disorders, autoimmune and inflammatory diseases and some types of cancer. Created with BioRender.com.

The human body is home to a myriad of bacteria that colonize its surfaces in the form of complex microbial communities called biofilms. In biofilms, bacterial cells adhere to a substrate and secrete an extracellular polymeric matrix (ECM) that holds the cells together and offers a defense against host immune responses and hostile environmental stresses.^1^ The ECM composition is variable and mostly comprises water and extracellular macromolecules, such as nucleic acids, polysaccharides, lipids, and proteins.^2^ The human gastrointestinal (GI) tract contains a rich and diverse microbiota, which density vary along its length tract, with the lowest numbers of microbes forming scattered biofilm fragments in the stomach and upper gut, whereas a rich and dense microbial biofilm in the large intestine.^3,4^ Many vital functions of the host are provided by the biofilm formed by the GI microbiota, including the maturation of the immune system, maintenance of the intestinal epithelial barrier, and resistance to pathogen colonization. However, not all microbial interactions promote health. Alterations in the GI microbiome, and consequently, the biofilm composition, termed dysbiosis, promote the release of pathobionts (normal resident microbes with pathogenic potential). Pathobionts contain virulence factors encoded by pathogenic islands, plasmids, and prophages, which are essential for colonization and replication within the host.^5^ These virulence factors may play a role in the onset and progression of various diseases, including: i) sepsis, where biofilm colonizing pathogens can subvert the immune system^6,7^ ii) colorectal cancer, where invasive bacterial biofilms modify epithelial barrier function and mucosal immunity to create a microenvironment conducive to carcinogenesis^8,9^ iii) inflammatory bowel diseases, where biofilm components from pathobionts stimulate the immune response and instigate or perpetuate microbial-driven inflammation^10,11^ and iv) neurological disorders, where biofilm components can initiate or exacerbate neurological diseases^12,13^ (Figure 1). Therefore, deciphering the biofilm composition of the gut microbiota can clarify the role of biofilm components in health or in the development of diseases.

In this review, I focus on the current knowledge of biofilm-associated proteins (BAP) in the gut microbiota and their potential role in neurodegeneration. Bacteria inhabiting the human gut produce BAP with the potential to form amyloids as scaffolds in the extracellular biofilm matrix. Genes encoding the BAP proteins of diverse bacteria are included in the accessory genome of the microbiome, suggesting that only certain strains would have the potential to produce BAP amyloids. Here, I discuss the importance of analyzing the genomic content of the microbiota rather than focusing on the presence of specific bacterial species. I also highlight the potential implications of BAPs as transmissible catalysts that facilitate the misfolding, accumulation, and spread of α-synuclein aggregates.

## Amyloid coding genes are enriched in gut microbiomes associated with neurodegeneration

In the upcoming decades more people be affected by neurodegenerative disorders, such as Parkinson’s disease (PD), Alzheimer’s disease (AD) and amyotrophic lateral sclerosis (ALS), especially as life expectancy increases.^14^ The high frequency of these incurable disorders is likely to have a devastating impact on individuals, families, and societies. These neurodegenerative disorders are characterized by progressive neuronal loss and dysfunction associated with the accumulation of aberrantly processed or misfolded proteins (amyloids), such as α-synuclein in PD, amyloid-β and hyperphosphorylated Tau in AD and TAR DNA binding protein 43 (TDP-43) in ALS. Misfolded proteins can also move between cells and seed the aggregation of their normal conformers in a “prion-like” spread of the pathology. The pathology of these conditions is also characterized by neuroinflammation produced by the pro-inflammatory cytokines produced both in the brain and periphery.^15^

PD is the second most common neurodegenerative disease worldwide; however, there are still no effective treatments to cure or stop its progression.^16^ A key pathological feature of PD is the presence of α-synuclein aggregates. However, the main cause of α-synuclein aggregation is unknown. Although there are rare hereditary cases, sporadic forms are responsible for the majority of cases (90%).^17^ Current evidence suggests that neurodegeneration begins in the enteric nervous system and propagates through the vagus nerve into the lower brainstem. This event occurs before the degeneration of dopaminergic neurons and onset of motor symptoms.^18,19^ Truncal vagotomy reduces PD risk.^20,21^ This is confirmed by several animal studies demonstrating that truncal vagotomy prevents the gut-to-brain spread of α-synucleinopathy.^22,23^ Therefore, considerable effort has been devoted to studying the changes that occur in the microbiota of patients with PD. Dysbiosis has been proposed as the etiopathology of PD^24–27^ Dysbiotic bacteria release structural components, neuroactive molecules and metabolites, including short chain fatty acids, lipopolysaccharide (LPS), bile acids, gamma-aminobutyric acid (GABA), and tryptophan precursors that may reach the brain via the bloodstream or enteric nervous system. This effect may intensify with age, as both the gastrointestinal tract epithelium and the blood-brain barrier become increasingly disorganized and permeable.^28^

Numerous studies have indicated that individuals with PD exhibit altered microbiomes, with the relative abundance of specific microbial species linked to the severity of functional symptoms ^24–26.27^ Transplantation of fecal microbiota from individuals with PD to α-synuclein-overexpressing mice lacking gut microbiota increases the severity of neurodegenerative symptoms compared to mice with microbiota from healthy controls.^29^ This experiment demonstrates that changes in the microbiome play an active role in neuropathological etiology, rather than merely being a consequence.^29^ Although the results of altered microbiota in the gut of patients with PD differ, certain findings have been replicated. A recent systematic review shows a consistent increase of *Verrucomicrobiaceae*, *Bifidobacterium*, *Alistipes*, *Christensenella*, *Enterococcus*, *Oscillospira*, *Bilophila*, *Desulfovibrio*, *Escherichia/Shigella*, *Akkermansia*, *Prevotella*, *Blautia*, *Faecalibacterium*, *Fusicatenibacter*, and *Haemophilus*, and decrease of *Lachnospiraceae*, *Prevotellaceae*, and *Pasteurellaceae* in patients with PD.^27^ However, no bacterial species have been identified as biomarkers for the differential diagnosis of PD.^30^

Most studies have been based on *16S* amplicon sequencing, revealing differences in the taxonomic composition between healthy controls and patients with PD. However, the emerging field of shotgun metagenomics enabled further investigations of all genetic material sampled from a community ^26,30–33^ Decoding complete metagenomes and cataloging their genes makes it possible to establish not only the presence or absence of a given bacterial species, but also its genetic diversity. A recent large-scale study based on deep shotgun sequencing of fecal DNA, which included 490 patients with PD and 234 control individuals, revealed that nearly 30% of species, genes, and pathways had altered abundance in PD.^26^ Reanalysis of the metagenomic data showed a strong correlation between the presence of genes encoding amyloids and PD. These include gene families related to curli in enterobacteria and BAP in diverse bacteria.^26,34^ Both the presence and abundance of these genes were enriched in the PD metagenomes, suggesting that the encoded amyloids may be linked to the mechanisms of PD.

## Gut microbiota produce BAPs with potential to form amyloids

Although exopolysaccharides and extracellular DNA are relevant and frequently essential compounds in the biofilm matrix, proteinaceous components also play a leading role in biofilm development. These proteins encompass a diverse range, including surface adhesins; proteinaceous subunits of cell appendages, such as pili, fimbriae, and flagella; proteins associated with outer membrane vesicles and amyloids.^2^ Among their functions, biofilm-related proteins are involved in cell attachment to surfaces; stabilization of the biofilm structure through interactions with exopolysaccharides and eDNA; development of a three-dimensional biofilm architecture; dispersion of the biofilm by enzymatically degrading polysaccharides, proteins, or nucleic acids.^35^

Amyloids are protein aggregates with a, mostly, organized β-sheet-rich structure.^36^ Many microbial species, including fungi and bacteria, secrete amyloids as components of the biofilm ECM. The most studied and characterized amyloid system produced by enteric bacteria is the curli. Curli fimbriae are composed of the major structural subunit, CsgA, and require the expression of a complex machinery encoded by the *csgBAC-csgDEFG* genes.^37,38^ A less complex group of amyloids has been described.^39^ These include cell surface proteins with amyloid modules that are processed and assembled into amyloid structures. One of the best-known examples is the BAP family, which is widespread in bacterial species.^40,41^ All BAPs have common characteristics; i) surface localization: BAPs are present on bacterial surfaces where they interact with the environment and other bacterial cells; (ii) size: BAPs are large proteins that often exceed 200 kDa; (iii) modularity: BAPs comprise multiple domains with different biological functions. They contain large and variable numbers of repeats with unknown functions; iv) function: BAPs promote cell-cell adhesion within biofilms and facilitate bacterial cell interactions. Many BAPs play important roles in infection. vi) Genome localization: *bap* genes are frequently located within mobile genetic elements.^40^

**Figure 2.**
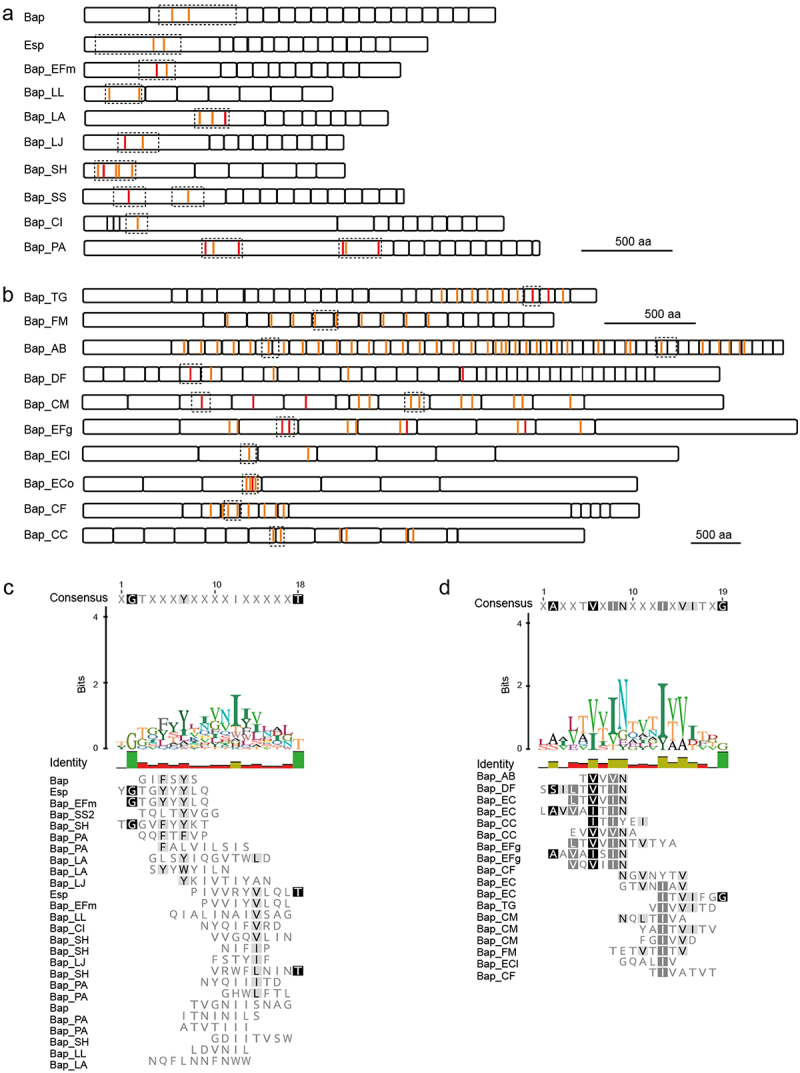
Schematic representation of BAPs. Colored lines represent aggregation-prone peptides. Red lines denote amino acid with amyloid features predicted by five (red) or four (orange) of the algorithms used. Dash boxes represent the amyloid regions. According to the localization of the amyloid peptides, BAP proteins can be divided into: (a) proteins with amyloid peptides at the N-terminal domain and (b) proteins with amyloidogenic peptides located at the core repeat domain. Alignment of the amino acids with aggregation propensity present at N-terminal domain (c) or located in the repeats (d) using Geneious Prime Software. BAP, biofilm-associated protein.

The Biofilm-associated protein (Bap) of *Staphylococcus aureus* was identified as the first member of the BAP family.^42,43^ Bap is a 238 kDa surface protein with a modular structure (Figure 2a), which is processed releasing the N-terminus to the media. The N-terminus comprises regions A (aa 45–360) and B (aa 361–818). The region A sequence was predicted to include coiled-coil motifs located at amino acids 226–308. Although it was initially thought that the coiled coil mediates biofilm formation through homophilic interactions between cells, the results showed that region A is dispensable for biofilm formation.^41^ However, the expression of a chimeric protein comprising region B in *S. aureus* lacking Bap is sufficient to induce aggregation and biofilm formation under acidic pH conditions.^41^ Several bioinformatics programs converged to predict several amyloid domains distributed in region B (Figure 2a). Two amyloidogenic peptides were characterized: peptide I _487_TVGNIISNAG_496_ and peptide II _579_GIFSYS_584_.^41^ These peptides, along with purified recombinant Bap B region, form fibrillar structures at low pH (<5) and bind amyloid-related dyes.^41^ Attenuated total reflectance-Fourier transform infrared spectroscopy analysis showed that aggregates are dominated by β-sheet/β-turn secondary structure, a feature of amyloid proteins. Region B comprised two active EF-hand calcium binding motifs. The binding of millimolar concentrations of calcium to EF-hand motifs affects Bap functionality by inhibiting biofilm formation.^44^ The crystal structure of the aggregation prone region of Bap shows that it adopts a dumbbell-shaped fold and exhibits tandem features.^45^ Experimental data showed that the Bap protein plays a dual role during biofilm development. First, Bap is expressed and covalently anchored to the cell surface. Then, Bap is processed releasing the N-terminus into the media. Under acidic conditions and low calcium concentrations, the N-terminus of the protein aggregates and self-assembles into amyloid-like structures, which mediate biofilm formation.^41,45^ Although the high number of repeats in the C region does not appear to play a direct role in biofilm formation, it has been proposed to be involved in strengthening the conformation of proteins on the cell surface, thus playing a role in the proper processing of the protein.^46^

The enterococcal surface protein (Esp) of *Enterococcus faecalis* is considered as a member of the BAP family because of its structural and functional homology with Bap of *S. aureus* (Figure 2a).^47,48^ The N-terminal domain of Esp (aa 49 to 743) shares 26% identity with region B of Bap and contains several amyloid domains.^47,49^ The purified recombinant N-terminal region of Esp forms aggregates with amyloid-like characteristics in acidic conditions (pH 4.2), as confirmed by biophysical analysis and binding to amyloid-related dyes.^49^ A low pH induces unfolding of the N-terminal region, leading to the aggregation and formation of amyloid-like structures that reinforce the biofilm architecture.^50^ The C-terminal repeats of Esp may project the N-terminal region outwards from the bacterial surface.^50^ Functionally, the presence of Esp has been correlated with the ability to form biofilm, as most of the *esp*-positive *E. faecalis* isolates form biofilm.^47,51^ Additionally, studies have suggested the existence of an Esp-independent pathway for biofilm formation.^52,53^

BAPs are widespread among bacteria, therefore, a recent study sought to identify BAP orthologs inhabiting the human gut.^34^ A series of BAP-like proteins have been identified through phylogenomic mining of sequenced gut microbiomes. These proteins fulfilled the criteria of the BAP family related to their high molecular weight and typical multidomain configuration. BAPs were found in abundance in Lactobacillales (*Lactococcus lactis*, *Lactobacillus acidophilus*, *Lactobacillus johnsonii*, *Enterococcus faecium* and *Streptococcus salivarius*), Enterobacterales (*Escherichia coli*, *Escherichia fergusonii*, *Citrobacter freundii*, *Enterobacter hormaechei*, *Chania multitudinisentens*, and *Providencia alcalifaciens*), Bacillales (*Staphylococcus hyicus* and *Terribacillus goriensis*), and Clostridiales (*Clostridium indolis* and *Finegoldia magna*).^34^ A rigorous computational analysis using the amyloid predicative algorithms WALTZ, AGGRESCAN, TANGO, MetAmyl, and FoldAmyloid, converged to detect amino acid stretches with amyloidogenic potential along the BAPs.^34^ The majority of BAP-derived domains formed amyloid-like structures, as evidenced by their expression in the curli-based export system, biophysical characterization, and binding of the purified recombinant domains to amyloid-indicative dyes (Table 1). Regarding amyloidogenic localization, two subgroups of BAP proteins were established: i) BAPs with predicted amyloid-prone domains in the N-terminal region (Figure 2a) and ii) proteins with amyloidogenic domains in the core repeats (Figure 2b). The first subgroup comprises BAPs from gram-positive bacteria, such as *L. lactis*, *L. acidophilus, L. johnsonii, E. faecium, S. salivarius, S. hyicus*, and *T. goriensis*, which mainly contain amyloid peptides in the N-terminal domain of the protein. This is a common feature of Bap and Esp, in which the N-terminal region is responsible for the amyloidogenic characteristics of the protein. There were no clear consensus sequences among the identified amyloid peptides (Figure 2c). This finding supports the notion that the ability to form amyloid fibril formation is an intrinsic property of polypeptide sequences. These proteins assemble into amyloid structures via proteolytic cleavage. Under specific environmental conditions, the released N-terminal domain that comprises the amyloid stretches and self-assembles into amyloid structures that mediate biofilm formation.^41^

**Table 1.** BAP proteins from the gut microbiota.

Amyloid features of the BAP proteins							
	Acc nº	Bacteria	Length (aa)	Nº repeats	Region tested for amyloid propensity (aa)^1^	Potential amyloid peptides from the region tested^2^	Characterization of the amyloid domains^3^
Bap	AAK38834.2	*S. aureus*	2276	14C	362–820	_487_TVGNIISNAG_496_ _579_GIFSYS_584_	CDAG (+); scattering (+) pH < 5; ThT (+) pH < 5; CR (+) pH < 5; ME (+)
Esp	VTT42696.1	*E. faecalis*	1953	7C	67–511	_386_PIVVYVLQLT_396_ _440_YGTGYYYLQ_448_	CDAG (+); scattering (+) pH4.2; ThT (+) pH4.2; CR (+) pH4.2; ME (+)
Bap_EFm	AGS76773	*E. faecium*	1732	6C	312–481	_398_PVVIYVLQL_407_ _452_GTGYYYLQ_459_	CDAG (+); scattering pH4.5 (+); ThT pH4.5 (+), pH7 (±); CR pH4.5 (+); ME pH4.5 (±)
Bap_LL	BAL51295.1	*L. lactis*	1372	5C	130–323	_137_QIALINAIVSAG_148_ _398_LDVNIL_407_	CDAG (+); scattering pH4.5/pH7 (+); ThT pH4.5/pH7 (+); CR pH4.5/pH7 (+); ME pH4.5/pH7 (+)
Bap_LA	AJP46937	*L. acidophilus*	1676	3B, 3C	585–810	_655_SYYWYILN_662_ _730_NQFLNNFNWW_739_ _798_GLSYIQGVTWLD_809_	CDAG (+); scattering pH4.5/pH7 (+); ThT pH4.5/pH7 (+); CR pH4.5/pH7 (+); ME pH4.5/pH7 (+)
Bap_SH	AJC96154	*S. hyicus*	1444	4C	70–270	_70_GDIITVSW_77_ _100_VVGQVLIN_107_ _169_TGGVFYYKT_177_ _188_VRWFLNINT_196_ _262_NIFIP_266_	CDAG (+)
Bap_LJ	CAX65971	*L. johnsonii*	1423	4B, 4C	220–530	_228_YKIVTIYAN_236_ _326_FSTYIF_331_	CDAG (+); scattering pH4.5 (+); ThT pH7 (±); CR pH7 (±); ME (-)
Bap_SS	MTR00538.1	*S. salivarius*	1720	10C	224–358	_243_SGYVYFTI_250_	CDAG (+)
					577–780	_583_TQLTYVGG_590_	CDAG (+); scattering pH4.5/pH7 (+); ThT pH4.5/pH7 (+); CR (-); ME (-)
Bap_CI	VDG65910	*C. indolis*	2313	2A, 7C	485–560	_540_NYQIFVRD_547_	CDAG (+); scattering pH4.5/pH7 (+); ThT pH4.5/pH7 (+); CR pH4.5/pH7 (+); ME pH4.5/pH7 (+)
Bap_PA	ATG15235.1	*P. alcalifaciens*	2520	9C	669–866	_670_QQFTFVP_676_ _706_ITNINILS_713_ _855_NYQIIITD_862_	CDAG (+)
					1428–1647	_1431_ATVTIII_1437_ _1450_GHWLFTL_1456_ _1630_FALVILSIS_1638_	CDAG (+)
Bap_FM	BAG07467	*F. magna*	2561	11B, 5C	1430	_1306_TETVTITV_1313_	CDAG (+); scattering pH4.5/pH7 (+); ThT pH4.5/pH7 (+); CR pH4.5/pH7 (+); ME pH4.5/pH7 (±)
Bap_AB	CAM85746.1	*A. baumannii*	7640	20B, 28 CD	1817–1988	_1938_TVVVNV_1943_	CDAG (-)
					5795–6004	_4791_VNGVNYTV_4798_	CDAG (-)
Bap_CM	AHG22648	*C. multitudinisentens*	6495	4A, 5B	1060–1294	_1208_NQLTIVA_1214_	CDAG (-)
					3322–3500	_3326_YAITVIT_3332_ _3430_FGIVVD_3435_	CDAG (-); scattering (-)
Bap_ECl	AJB81761.1	*E. hormaechei*	6017	5B	1602–1768	_1688_GQALIV_1693_	CDAG (+); scattering pH4.5 (+); ThT pH4.5 (+), pH7 (±); CR pH4.5 (+); ME pH4.5 (±)
Bap_ECo	AHG13161.1	*E. coli*	5662	6B	1640–1770	_1647_LTVVIN_1652_ _1681_GTVNIAV_1687_ _1707_LAVVAITIN_1715_ _1747_ITVIFGG_1753_	CDAG (+)
Bap_CF	AHY14400	*C. freundii*	5630	6B, 4C	1440–1583	_1448_VQVIIN_1453_ _1575_TIVATVT_1581_	CDAG (+); scattering pH4.5 (+); ThT pH4.5 (+), pH7 (±); CR (-); ME pH4.5 (±)
Bap_CC	EAT97336	*C. concisus*	5080	4B, 3C	1929–2019	_1944_ITIYEI_1949_ _2000_EVVVVNA_2006_	CDAG (-)

^1^Fernández-Calvet, A., Matilla-Cuenca, L., Izco, M., Navarro, S., Serrano, M., Ventura, S., et al. (2024). Gut microbiota produces biofilm-associated amyloids with potential for neurodegeneration. Nature Communications, 15(1), 4150–19.

^2^Amino acid stretches predicted to be amyloidogenic by at least four out of the five algorithms tested. Amyloid predicted algorithms: WALTZ, AGGRESCAN, TANGO, MetAmyl, and FoldAmyloid.

^3^CDAG: Expression of the amyloid regions in the pExport of the Curli-dependent amyloid generator system, CDAG (+): Congo red binding positive. CDAG (-): Congo red binding negative. Scattering: Synchronous light scattering of recombinant BAP amyloids incubated at pH 4.5 or pH 7, measured exciting at 330 nm, and recording in the range of 320 − 340 nm. ThT: Thioflavin-T binding of recombinant BAP amyloids, incubated at pH 4.5 or pH 7. Th-T emission fluorescence was detected using an excitation wavelength of 445 nm and recording in the range of 460–600 nm. CR: Congo red binding of recombinant BAP amyloids, incubated at pH 4.5 or pH 7. Optical absorption spectra were recorded from 375 to 700 nm. ME: Transmission electron microscopy of recombinant BAP amyloids, incubated at pH 4.5 or pH 7. ME (+): fibrillar structures detected. ME (±): protofibrillar structures detected.

The second group of BAPs included extremely large proteins, in which the predicted amyloidogenic peptides were located at the core repeat domain (Figure 2b). These BAPs are mainly found in gram-negative bacteria (*A. baumannii*, *E. coli*, *E. fergusonii*, *C. freundii*, and *E. hormaechei*), but also in gram-positive bacteria, such as *S. epidermidis*, which has an amyloid peptide (STVTVT) repeated 17 times in the C-terminal region.^54^ Sequence analysis of the amyloid stretches revealed the potential consensus sequences, AxxTVxIN and xIVVIxxG (Figure 2d). The presence of amyloid-prone domains within tandem repeats may promote interactions between repeating units, enabling the formation of fibrillar structures and the regulation of their stability.^46^ However, the precise mechanism underlying amyloid formation requires further experimental analysis and validation.

## Bap-genes are part of the accessory genome of the microbiota

The concept of the “pan-genome” in which strains of the same species share a core set of genes and accessory genes are found only in a subset of the strains, is of substantial interest. Advances in sequencing technology have led to the application of microbial pan-genome and metagenomic analyses to complex communities.^55,56^ Therefore, detecting whether biofilm-specific genes, such as *bap* genes, are shared by all members of a community is of great interest. A search analysis of *bap* genes among multiple genome sequences of diverse bacteria showed that 70% of the *bap* genes are clear accessory genome components, indicating that they are only present in some, but absent in other strains of the same species (Table 2). The analysis showed that *bap* genes are located in mobile genetic elements, such as transposons, pathogenicity islands, and plasmids.

**Table 2.** Prevalence of *bap* genes in whole-genome sequences.

Bacteria	Gene	Total genomes	Genomes *bap*-positive	*bap-*positive %	Plasmids *bap*-gene positive
*S. aureus*	*bap*	1077	6	1	–
*S. epidermidis*	*Bap_SE*	206	5	2	3
*E. faecalis*	*esp*	641	197	31	5
*E. faecium*	*bap_EFm*	408	224	55	1
*L. johnsonii*	*bap_LJ*	18	4	22	–
*S. salivarius*	*bap_SS*	20	3	15	–
*C. concisus*	*bap_CC*	16	6	38	–
*E. coli*	*Bap_ECo*	1761	352	20	–
*L. lactis*	*bap_LL*	84	79	94	–
*E. cloacae*	*bap_ECl*	377	371	98	–
*A. baumannii*	*bap_AB*	767	755	98	3
*L. acidophilus*	*bap_LA*	23	23	100	–
*E. fergusonii*	*bap_EFg*	74	74	100	–

**Figure 3.**
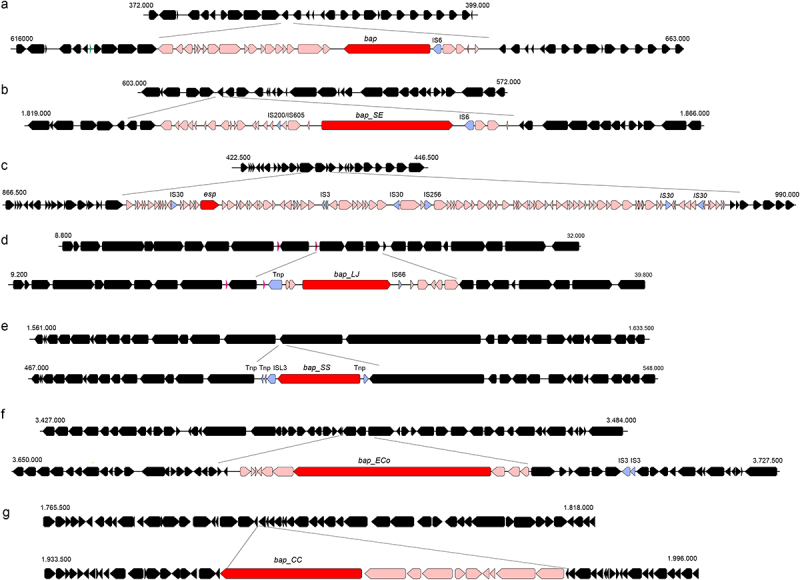
BAP coding genes are located in mobile genetic elements. Alignment of genome regions comprising BAP coding genes. Colored sections indicate the modules comprising *bap* genes. BAP genes are shown in dark red. Transposase (Tnp) and insertion sequences (IS) coding genes are shown in blue. a) *Staphylococcus aureus* 8325 and *S. aureus* V329 (*bap* +). b) *Staphylococcus epidermidis* RP62A and *S. epidermidis* 7049 (*bap_ep* +). c) *Enterococcus faecalis* D32 and *E. faecalis* 62 (*esp* +). d) *Lactobacillus johnsonii* N6.2 and *L. johnsonii* FI9785 (*bap_lj* +). e) *Streptococcus salivarius* JIM8777 and *S. salivarius* SALI-10 (*bap_ss* +). f) *Escherichia coli* M217 and *E. coli* THO-008 (*bap_eco* +). g) *Campylobacter concisus* ATCC33237 and *C. concisus* 13826 (*bap_cc* +).

The first example is *S. aureus* V329. The Bap protein of *S. aureus* is carried by a transposon that forms part of the 27 kb mobile pathogenicity island, SaPIbov2^57^ (Figure 3). SaPIbov2 contains 24 open reading frames and is delimited by direct 18 bp sequence repeats. The module comprising the *bap* gene also includes genes encoding ABC transporters and transposases. This module substitutes for the toxin genes present in other pathogenicity islands of *S. aureus*. SaPIbov2 is mobilized by the activity of a functional recombinase belonging to the integrase family, Sip, which promotes excision, circularization and site-specific integration at the *att*_*B*_ site of the *S. aureus* chromosome.^57^ In *S. aureus* V329, the transposase encoded by the *bap* transposon is inactive, maintaining *bap* stability on the chromosome.^57^ Some isolates of the closely related species *S. epidermidis*, carry a *bap* ortholog gene, which is carried in a transposon and forms part of a mobile pathogenicity island (Figure 3). However, in other staphylococcal species, such as *Staphylococcus warneri* and *Staphylococcus saprophyticus*, *bap* genes are part of the core genome.^58^ This suggests that the product encoded by the *bap* gene may play adaptive functions depending on the staphylococcal species and specific environments they occupy.

The *E. faecalis esp* gene is located within a 153 Kb pathogenicity island flanked by 10 bp direct repeats^59^ (Figure 3). Pathogenicity island include genes encoding virulence factors (cytolysins, hydrolases, surface proteins, and general stress proteins), transposases, transcriptional regulators, integrases, and excisionases. Certain variations have been detected in the region of the island encoding the *esp* gene, which include a 17 kb deletion in the strain *E. faecalis* V583.^59^ In *E. faecium*, a *bap-*like gene is inserted into a pathogenicity island, which differs from the *esp*-containing pathogenicity island of *E. faecalis*.^53^ It also contains two putative virulence genes that encode NADH oxidase, muramidase, and autolysin.^53^ Although the presence of the Esp island is widespread among *E. faecalis* and *E. faecium* clinical isolates, it does not appear to be a strict prerequisite for survival in the hospital environment, as none of the genes of the pathogenicity island are completely present in hospital-associated clusters.^60^

Comparative analysis of the genomes of *L. johnsonii* and *S. salivarius* showed that *bap* genes belong to the accessory gene repertoire and are associated with mobile elements, such as insertion sequences and transposases (Figure 3). The *bap*-carrying modules also included hypothetical proteins with unknown functions. However, in bacteria such as *E. coli* and *Campylobacter concisus, bap* is not located adjacent to apparent transposable elements as they are not flanked by insertion sequences or other mobile element markers (Figure 3). Notably, the *bap* gene can be detected in virulence plasmids of different bacteria, such as *S. epidermidis*, *E. faecalis*, *E. faecium*, and *A. baumannii*, which reinforces the concept that *bap* genes can be horizontally transferred between bacteria through processes, such as conjugation, transformation, or transduction.^61^ Therefore, it will be of great interest to determine the transmissibility of *bap* genes among members of the gut microbiota, as well as to identify the existence of factors, such as pH or inflammatory environment that promote their spread.

## Bap-amyloids are naturally produced at the human gut

Amyloid-encoding genes are part of the intestinal metagenome.^26,34^ However, the presence of amyloid fibers in enteric biofilms has been underestimated. Sequencing data provided information on the genetic load of the sample. However, the presence of these genes did not guarantee protein expression. Even if a protein is expressed, it does not indicate the formation of amyloid structures. Notably, BAPs are facultative amyloids.^62^ They are secreted in a functional globular folded state, and require precise environmental conditions to modulate their conformation and form an amyloid fold. Therefore, neither the presence of the gene nor the protein guarantees the formation of amyloid structures.

To detect amyloids in the intestinal biofilm, the insoluble material of the human fecal microbiota of individuals with irritable bowel syndrome (IBS) was purified.^34^ Patients with IBS normally have a dysbiotic intestinal microbiota, intestinal barrier dysfunction, and a greater risk of developing PD than healthy patients ^63–65^ Sodium dodecyl sulfate-insoluble aggregates purified from fecal samples were detected using anti-BAP-specific antibodies.^34^ Moreover, the purified aggregates were susceptible to formic acid and trifluoroacetic acid/hexafluoroisopropanol treatments, and reacted with the amyloid conformational antibodies, A11 and OC, validating the amyloid nature of the BAP aggregates purified *in vivo*.^34^

Other studies have also supported the presence of amyloids in the human gut. Christensen et al. identified two proteins with amyloid properties in the microbiomes of PD transgenic rats using differential solubilization of proteins at high concentrations of formic acid and mass spectrometry.^66^ Pr12 and Pr17 are produced by *Prochlorococcus marinus* and *Candidatus Thiomargarita nelsonii*, respectively. Although these organisms are aquatic, the authors proposed that homologs of Pr12 and Pr17 proteins could be present in other microorganisms whose genomes are not yet available. Notably, none of the identified sequences showed considerable similarity to CsgA.^66^

Whether curli amyloids are expressed by enteric bacteria in the gut is debatable as there is only indirect evidence of their expression in vivo.^67–70^ Oral infection of mice with Salmonella enterica serovar Typhimurium results in the expression of curli in the intestinal tract.^67^ The amyloid nature of curli was confirmed by depolymerization of amyloids using 90% of formic acid. Other studies have reported the presence of curli proteins in the feces of humans and rats; however, the amyloidogenic properties of the detected curli have not been investigated.^71^

All these data support the existence of a diverse composition of amyloids in the gut microbiome, “the amylome.” However, discovery, mapping, and deeper characterization of the full human gastrointestinal amylome need to be performed.

## BAPs as molecular mimicry molecules affecting human amyloids

Based on accumulating knowledge on microbiome-associated amyloids and the likely active role of the microbiome in neurodegenerative diseases, host exposure to the amylome may present a key opportunity to trigger disease through mechanisms of molecular mimicry. Although there is a wide variety of amyloid fiber architectures, bacterial amyloids are considered canonical molecular mimics of human amyloids^72–74^ This is reflected by the fact that bacterial amyloid intermediates react with amyloid conformational antibodies that recognize human amyloids.^34,37,75^

Two mechanisms of molecular mimicry are considered: i) bacterial amyloids may cause inflammation by priming the innate immune system, which involves the chronic activation of immune cells, induction of pro-inflammatory cytokines, and aggregation of human amyloids; ii) bacterial amyloids can polymerize fibrils of certain non-homologous amyloids in a process called cross-seeding, by acting as aggregation nuclei.

Reactive oxygen species (ROS) is emerging as a key detrimental factor of age-related neurodegenerative diseases. It has been reported that bacterial cell-wall components such as peptidoglycan, LPS and toxins translocated to the brain may induce mitochondrial ROS production and activation of the NF-κB pathway triggering inflammatory responses and neuronal death^76–78^. Therefore, it is possible that bacterial amyloids from the gut may act as canonical molecular mimics of human molecules by priming an inflammatory response and altering the levels of ROS.^13,74,79,80^ This is the case of curli of enterobacteria and human amyloid Aβ, both of which stimulate Nos2 production (a hallmark of inflammation) in macrophages and microglia cells through a TLR2-dependent mechanism^81–83^ However, the lack of a neuroinflammatory response in the substantia nigra pars compacta of mice after the interstitial injection of BAP amyloids suggest that it is not the main mechanism by with BAP amyloids induce α-synuclein aggregation.^34^

**Figure 4.**
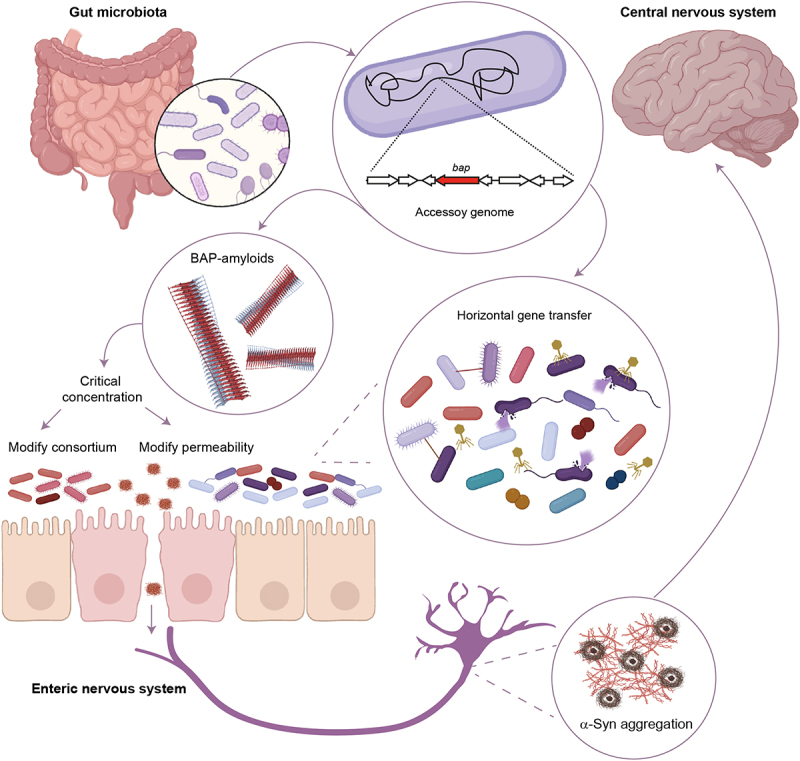
BAP amyloids of the gut microbiota and their role in neurodegeneration. Bacteria inhabiting the human gut produce biofilm-associated proteins (BAP) with potential to form amyloids. bap-coding genes are encoded in the accessory genome of the microbiome, with potential for horizontal transfer between species. This fact introduces a level of microbiome variability, which is not limited to the presence of specific bacterial species but to a specific gene cargo. A threshold concentration of BAP amyloids may have collateral effects on the intestine by disturbing the intestinal permeability or by modulating the physiology of the consortium of gut bacteria. BAP amyloids translocated from the gut may exert their effects on the central nervous system through the enteric nervous system. BAP amyloids colocalize with α-synuclein (α-Syn) catalyzing their aggregation and increasing α-Syn half-life. Created with BioRender.com.

Cross-kingdom interactions of enteric amyloids have been mostly limited to studies on curli/CsgA from Enterobacteriaceae^84–86^ However, a recent study extends the literature to include potential capabilities of the BAP amyloids in the interaction with α-synuclein.^34^ BAP amyloids colocalize with α-synuclein expressed by human-derived dopaminergic cells and seed α-synuclein aggregation *in vitro* and animal models.^34^ Moreover, BAP amyloid exposure increases the α-synuclein half-life, which is associated with a decrease in chaperone-mediated autophagy activity, which is associated with PD.^87,88^ The mechanism by which this process affects subsequent amyloid aggregation in the brain remains unknown. The gut-brain axis can be considered a connection bridge between amyloids from the gut and the brain. Transmission from the gut to the brain has been described for prions in transmissible spongiform encephalopathy.^89^ Upon oral ingestion, prions survive the digestive process and are incorporated into the gut. In the intestinal epithelium, prions interact with immune cells and accumulate in the follicular dendritic cells and other lymphoid follicles. Prions then move to the enteric nervous system and finally spread to the central nervous system (CNS).^89^ Similar spreading mechanisms may be responsible for the propagation of other amyloidogenic proteins that cause neurodegenerative disorders ^90–92^ Aligned with this hypothesis, pathological studies on patients with PD have shown remote locations of α-synuclein aggregates, such as the enteric nervous system, the olfactory bulb and dorsal motor nuclei of the vagus nerve, suggesting that α-synuclein aggregation might initiate at the neuronal cells of the olfactory bulbs and/or the gut and progresses from these locations into the CNS.^19^ Considering that BAP amyloids exist in the gut, BAP amyloids may recruit soluble α- synuclein monomers in the peripheric areas and seed their aggregation initiating a prion-like spread of pathologic α-synuclein (Figure 4). Similarly, intestinal colonization of mice overexpressing α-synuclein with curli-producing bacteria may promote α-synuclein pathology in the gut and the brain.^76^ Additionally, the threshold concentration of bacterial amyloids may have collateral effect on the intestine. They can modulate the physiology of the microbial consortium by inducing changes in the bacterial composition or by promoting inter-species amyloid aggregation. However, disturbing intestinal barrier permeability favors the contact of bacterial amyloids with α-synuclein or their spread to the CNS.

## Conclusions and remarks

The intricate interplay between the microbiome and host presents a multifaceted understanding of environmental and genetic factor influence on neurodegenerative diseases.

Current data suggest that PD involves not only genetic and environmental factors, but also transmissible mechanisms. They include the propagation of α-synuclein aggregated forms from cell to cell, inducing further misfolding in neighboring neurons through mechanisms similar to prion-like transmission. In the misfolding of normal to toxic α-synuclein, aggregated forms can start at the enteric nervous system, where the microbiome plays a relevant role. Then, α-synuclein spreads to the brain via the vagus nerve. However, accessory genomes and mobile genetic elements further add to this complexity by promoting genomic variability, which may contribute to neurodegeneration. The accessory genome of the microbiome encodes BAP-coding genes. This introduces a level of microbiome variability that is not limited to the presence of specific bacterial species, but to a specific gene cargo. In addition, the fact that genes coding for BAP-amyloids are located in mobile genetic elements suggests that transposable elements may play a substantial role in the origin and progression of PD by disseminating BAP-coding genes among bacterial communities. The intestinal biofilm, where bacterial densities are high and mobile elements are frequent, provides an environment conducive to gene exchange, enhancing genetic diversity and the spread of virulence factors that allow bacteria to adapt to different environments; however, this could have disastrous consequences for the host. Therefore, the presence of BAP amyloids in the accessory genome and association with mobile genetic elements may be considered as transmissible catalysts that promote the misfolding, accumulation, and spread of α-synuclein aggregates. Understanding the complex interactions between the microbiota, transmissible elements, and host is crucial for developing novel therapeutic approaches to combat microbiota-related diseases and improve overall health outcomes. The possibility of modulating the gut microbiome and/or amyloidogenesis (amyloid polymerization) and stop the dissemination of BAP-coding genes among bacterial communities, can be considered as therapeutic options to prevent or reduce the risk of neurodegenerative diseases. These applications could even lead to a personalized medicine, considering *bap* cargo an individual’s amyloid composition at given the time of the treatment. This field of microbiome research carries enormous clinical translational potential and will help improve the life quality of older adults.

## Data Availability

Data on BAP-genes and accessory genome is available on request from the authors
